# Functional studies in a neonate with a DOCK11 VUS guide clinical care

**DOI:** 10.70962/jhi.20260053

**Published:** 2026-06-30

**Authors:** Patrick O’Connell, Conor Gruber, Sofija Buta-Panov, Jaya Ganesh, NY-ROYAL Consortium, Dusan Bogunovic, Joshua Milner, Xiao Peng, David Beck, Noelle Lynch, Charlotte Cunningham-Rundles, Carrie Lucas, Shen-Ying Zhang, Sofija Buta Panov, Joseph Oved, Letticia Bright, Bertrand Boisson, Dusan Bogunovic

**Affiliations:** 1 Department of Genetics and Genomic Sciences, Icahn School of Medicine at Mount Sinai, New York, NY, USA; 2 Department of Pediatrics, Icahn School of Medicine at Mount Sinai, New York, NY, USA; 3 Department of Pediatrics, Columbia University Medical Center, New York, NY, USA; 4 Center for Genetic Errors of Immunity, Columbia University Medical Center, New York, NY, USA

## Abstract

O’Connell et al. demonstrate that negative functional studies can meaningfully guide care. In a neonate with a DOCK11 variant of uncertain significance, negative functional studies guide clinical care and highlight the broader value of publishing instructive negative findings in immunology and genomic medicine.

## Introduction

Dedicator of cytokinesis 11 (DOCK11)–autoinflammatory disorder is an X-linked, monogenic disorder of immune regulation caused by hemizygous loss-of-function variants in DOCK11 (1, 2). Clinically, it most commonly presents in childhood with a spectrum that includes recurrent infections, persistent systemic inflammation with fevers and elevated acute-phase reactants, variable cytopenias, benign lymphoproliferation, and organ-specific inflammatory manifestations such as enterocolitis or panniculitis. The 13 cases reported to date demonstrate a highly heterogeneous phenotype, but a consistent pattern of immune dysregulation and susceptibility to both infectious and autoimmune complications.

At the cellular level, DOCK11 functions as a guanine-nucleotide exchange factor that regulates cell division cycle 42 (CDC42)–dependent actin cytoskeletal dynamics, and it has been shown that loss-of-function of DOCK11 impairs CDC42 signaling and downstream pathways (including signal transducer and activator of transcription 5 (STAT5) activation, filopodia formation, immune cell migration, and regulatory T cell phenotypes), producing an “immune-related actinopathy” that links cytoskeletal defects to immune dysregulation. Genotype–phenotype correlations are emerging: variants that abolish DOCK11 expression tend to associate with a more autoinflammatory, infection-prone phenotype (recurrent pneumonias and systemic inflammation), whereas certain missense alleles more commonly present with autoantibody-mediated autoimmunity (1). A number of the most severely affected patients have died in early childhood from infectious and inflammatory complications (2). Given the severe presentations of DOCK11-autoinflammatory disorder, hematopoietic stem cell transplant (HSCT) has been proposed, but there are no published reports of this having been performed to date.

## Case presentation

**Figure 1.**
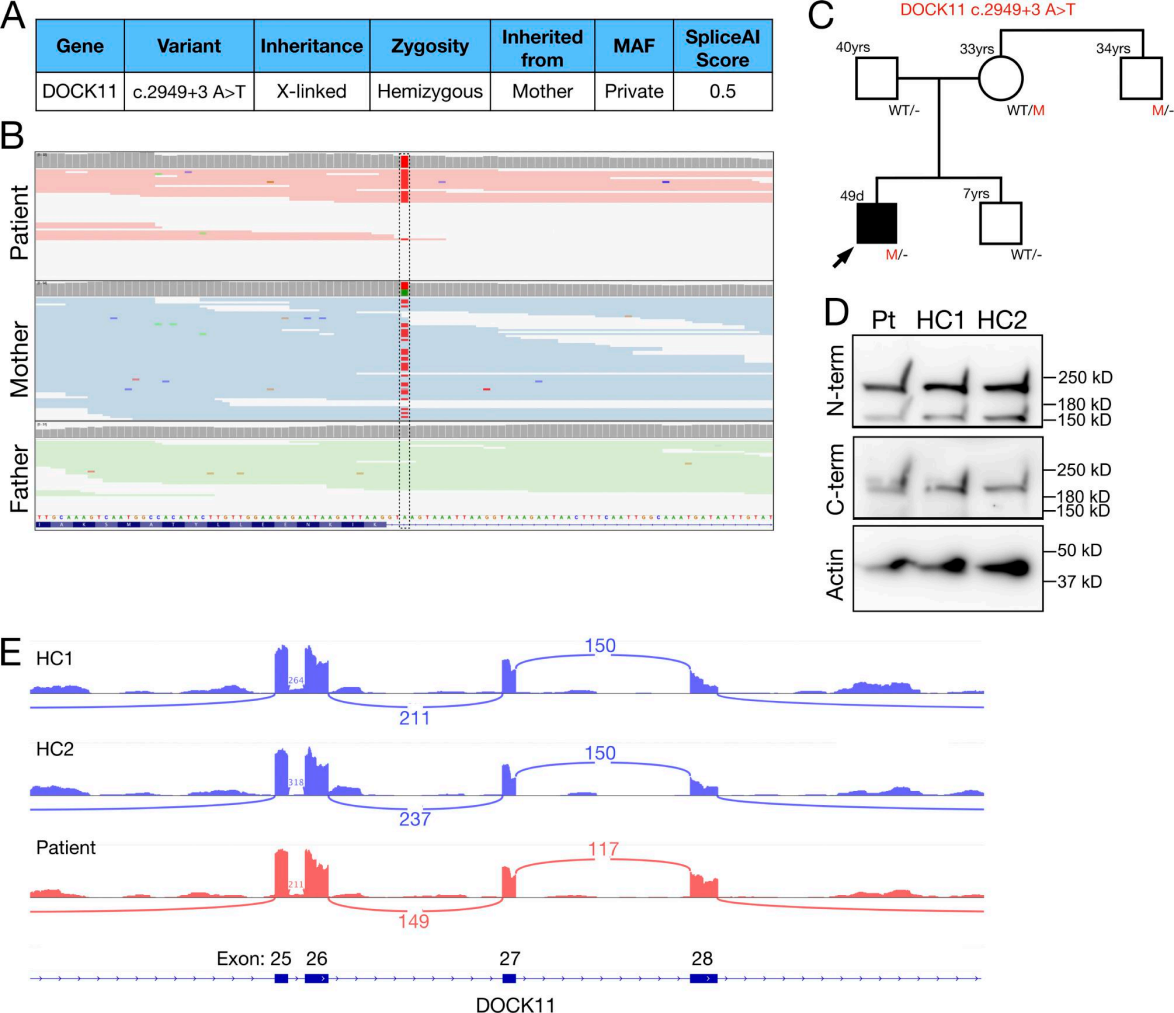
**Functional evaluation of a DOCK11 VUS. (A)** Table with characteristics of the DOCK11 variant. **(B)** Pile-up of WGS reads for the patient, mother, and father. **(C)** Pedigree of the affected patient. Genotype is listed for each individual, and the presence of the mutant allele is indicated by a red “M.” **(D)** Western blot of T cell blasts from the patient and two unrelated healthy controls (HC). Top panel: antibody to N terminus of DOCK11 showing normal protein at the expected size of DOCK11 (240 kD). Middle panel: antibody to C terminus of DOCK11 also with a band at expected size for DOCK11. Equivalent expression of DOCK11 noted as compared to both healthy controls. **(E)** Sashimi plot of DOCK11 from RNA-seq of whole blood. Normal read coverage on exon 27 and normal splicing pattern appreciated in patient compared to controls. The patient’s variant is located just 3′ to exon 27 and was predicted to lead to exclusion of this exon if pathogenic. All experiments were performed once with two healthy control samples. Source data are available for this figure: SourceData F1.

We present a 49-day-old, full-term neonate who came to our attention as a transfer to our hospital for evaluation of acute liver failure (ALF) of unknown etiology. The patient presented initially a few days earlier with recurrent non-bilious non-bloody emesis, dark urine, and hematochezia. Evaluation revealed aspartate aminotransferase (AST) (856 U/L) and Alanine Aminotransferase (ALT) (686 U/L), Gamma-Glutamyl Transferase (GGT) of 259 U/L, total bilirubin of 5.6 mg/dl, International Normalized Ratio (INR) of 2.5, normal C-reactive protein, gallbladder edema, thrombocytopenia (146 × 10^3^/μl), and an *Escherichia coli* urinary tract infection (UTI). He was evaluated for liver transplant given his acute cholestatic hepatitis at our center, during which time his liver function worsened with a peak INR of 7.6. He underwent orthotopic liver transplant (OLT) immediately, which was complicated by bowel dehiscence, multiple obstructive blood clots in the bladder, hemidiaphragm collapse, anemia, multiple episodes of acute kidney injury, and a second *E. coli* UTI. On exam, the patient was noted to have mild hepatosplenomegaly, scleral icterus, scrotal edema, and abdominal distension. Rapid trio whole-genome sequencing (WGS) was sent, which revealed a single variant of uncertain significance (VUS) in DOCK11 (Fig. 1, A and B).

This DOCK11 VUS was a maternally inherited splice site variant, not found in gnomAD, and with a SpliceAI score of 0.5 (Fig. 1, A and B), which, together with this patient’s phenotype of ALF, hepatosplenomegaly, recurrent *E. coli* infections, anemia, and thrombocytopenia, suggest a possible molecular explanation for the patient’s presentation. The SpliceAI score of 0.5 falls just within the possible pathogenic threshold, which further supports why this VUS was reported by the clinical lab (3). While ALF is not a known phenotype of DOCK11-autoinflammatory disorder, one must consider that the current phenotype is likely incomplete given the limited number of cases reported and that ALF is a complication common to many inborn errors of immunity (IEIs). We first re-examined our patient’s phenotype and noted marked poikilocytosis and anisocytosis of RBCs as well as giant platelets, all of which can be seen in actinopathies (4). Furthermore, biopsy of the native liver showed extensive extramedullary hematopoiesis (high CD68 and CD163 staining), hemosiderin-laden macrophages, and occasional hemophagocytes, consistent with a pathological diagnosis of macrophage activation syndrome.

Considering the severe illness in this patient, the multiple postoperative complications, and the fact that DOCK11-autoinflammatory disorder is a combined immunodeficiency (CID) that has resulted in childhood mortality, we decided to pursue familial and functional studies to determine if this DOCK11 VUS was pathogenic and an HSCT evaluation was warranted.

We first assessed if the DOCK11 variant segregated within the family. The patient’s healthy, 7-year-old brother was tested and did not carry the DOCK11 VUS (Fig. 1 C). Additionally, the patient’s healthy, 34-year-old maternal uncle was tested and he did carry the DOCK11 VUS (Fig. 1 C). Presumably by the age of 34, some phenotype of DOCK11-autoinflammatory disorder would have presented by now, but given the propensity for incomplete penetrance in IEIs, this alone was not enough rule out pathogenicity of this VUS. To further evaluate this VUS, we generated T cell blasts from the patient and two unrelated healthy controls and performed a western blot for DOCK11. Antibodies to both the N- and C-terminus were used to rule out the potential for a partial protein product as this variant is located in the center of the DOCK11 protein. Approximately equivalent expression of DOCK11 was detected in the patient compared to the controls using both antibodies (Fig. 1 D). While this finding argued against the potential for this splice site variant to affect protein production, multiple DOCK11 patients with missense mutations have normal DOCK11 protein expression in T cell blasts (1, 2). Of note, however, the only reported splice site variant and two other frameshift variants in DOCK11 are known to reduce DOCK11 protein expression in T cells (1, 2, 5). To definitively rule out the possibility that this variant is pathogenic, we performed RNA sequencing (RNA-seq) on whole blood from the patient and two healthy controls (Fig. 1 E). Splicing was assessed via sashimi plots at exon 27 (where this variant was predicted to induce exon skipping if pathogenic), and we confirmed no aberrant splicing in the patient compared to controls (Fig. 1 E). Together, these findings led us to rule out DOCK11-autoinflammatory disorder as a cause for this patient’s symptoms.

## Conclusion

VUS resolution in genes known or suspected to cause an IEI presents particularly challenging situations given the high rate of incomplete penetrance, variable expressivity, and often incompletely described phenotypes among this class of conditions. Confirming the pathogenicity of a genetic variant is critical in many IEIs, particularly those causing a severe CID, as the decision to pursue HSCT is not one to be taken lightly. In this case, we demonstrate our workup for a VUS in a recently described IEI gene, which resulted in our reclassification of this DOCK11 variant as likely benign and removed discussion of HSCT for this patient.

It could be argued that our workup was too extensive and that the SpliceAI score of 0.5 was not high enough to raise true concern for a splicing defect and that once normal DOCK11 protein expression was noted in T cells, there was no need for further evaluation. To the first point, we chose to pursue this workup given the strong fit of the patient’s phenotype with DOCK11-autoinflammatory disorder and considering the sensitivity and validation rate for a SpliceAI score of 0.5 is 0.6 and ∼0.55, respectively (3). To the second point, given that only a single DOCK11 patient with a splice site variant has been described and considering that the exon predicted to be potentially excluded was only 3 kD and in-frame, we could not exclude the possibility of a nonfunctional protein product, which was undetectable on standard western blot.

On follow-up at 15 months of age, this patient has had no further liver or immunological concerns and is growing and developing well with only mild developmental delays. Thus far, no other cause has been identified for his ALF and a research-level investigation of his WGS by our team was unrevealing (data not shown). Given our institution is a large pediatric liver transplant center, it is not uncommon for us to come across cases of pediatric ALF with a completely negative workup including WGS. This is an area where more research is certainly needed including studies to look for potential genes of uncertain significance and the novel syndromes they may cause. This case demonstrates challenges geneticists and immunologists are increasingly facing in the era of genetics-first clinical evaluations of complex patient presentations and the stepwise approach we took to solve this specific case.

## Ethics declaration

The patient’s family provided informed consent under an institutionally approved institutional review board (Mount Sinai IRB).

## Supplementary Material

SourceData F1 is the source file for Fig. 1.

## Data Availability

Data sharing is limited given it is classified as protected health information. Contact the corresponding author for requests for raw data. Additional clinical information available from corresponding author upon request. This variant has been submitted to ClinVar under Variant ID: 3897941 and Accession: VCV003897941.2.
