# Supplementary figures and images for: Functional studies in a neonate with a DOCK11 VUS guide clinical care
Source: J Hum Immun. 2026 Jun 30;2(5):e20260053. doi: 10.70962/jhi.20260053 (PMC13317482; doi:10.70962/jhi.20260053)

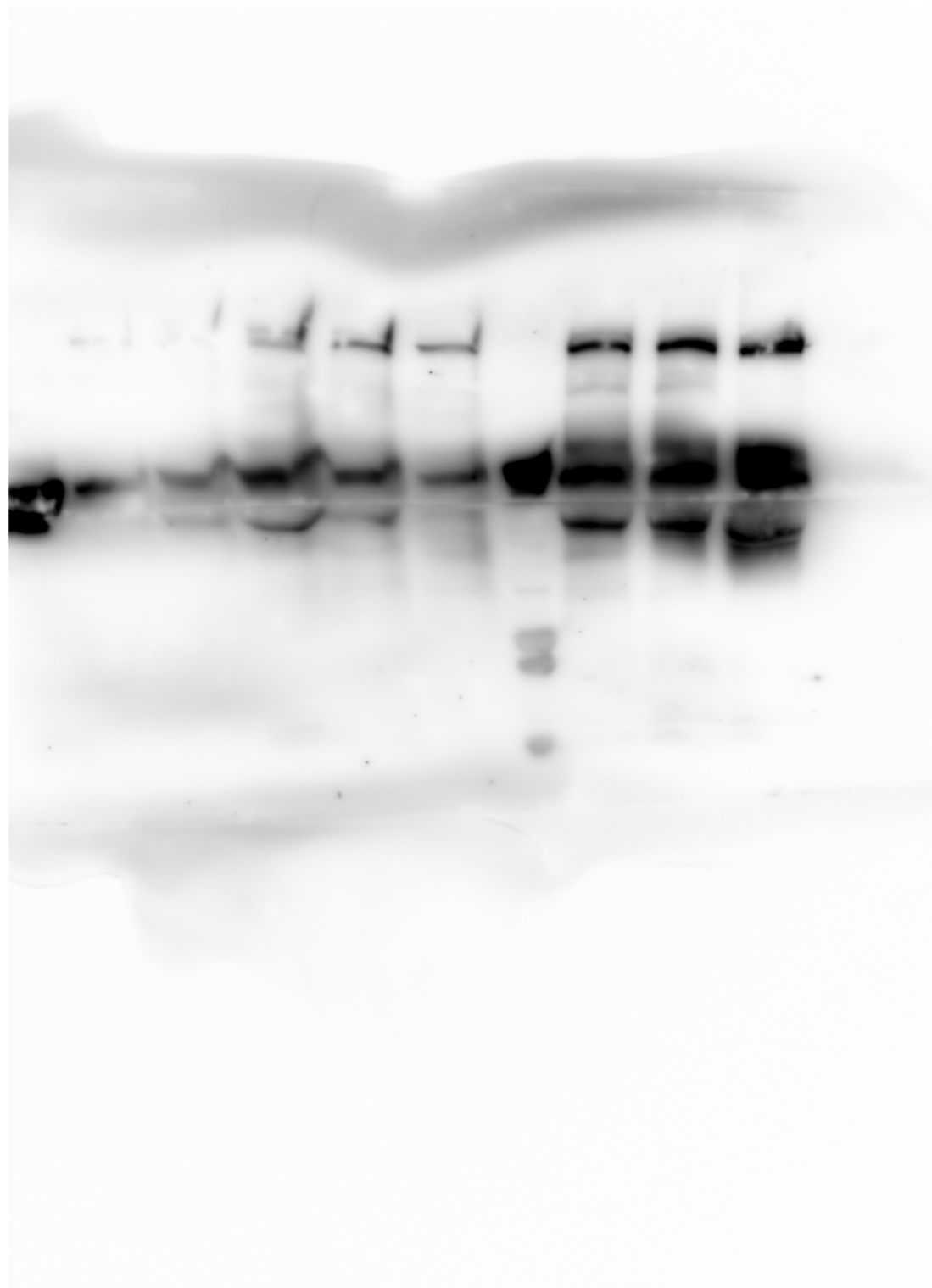

nterm DOCK11 T cells WB

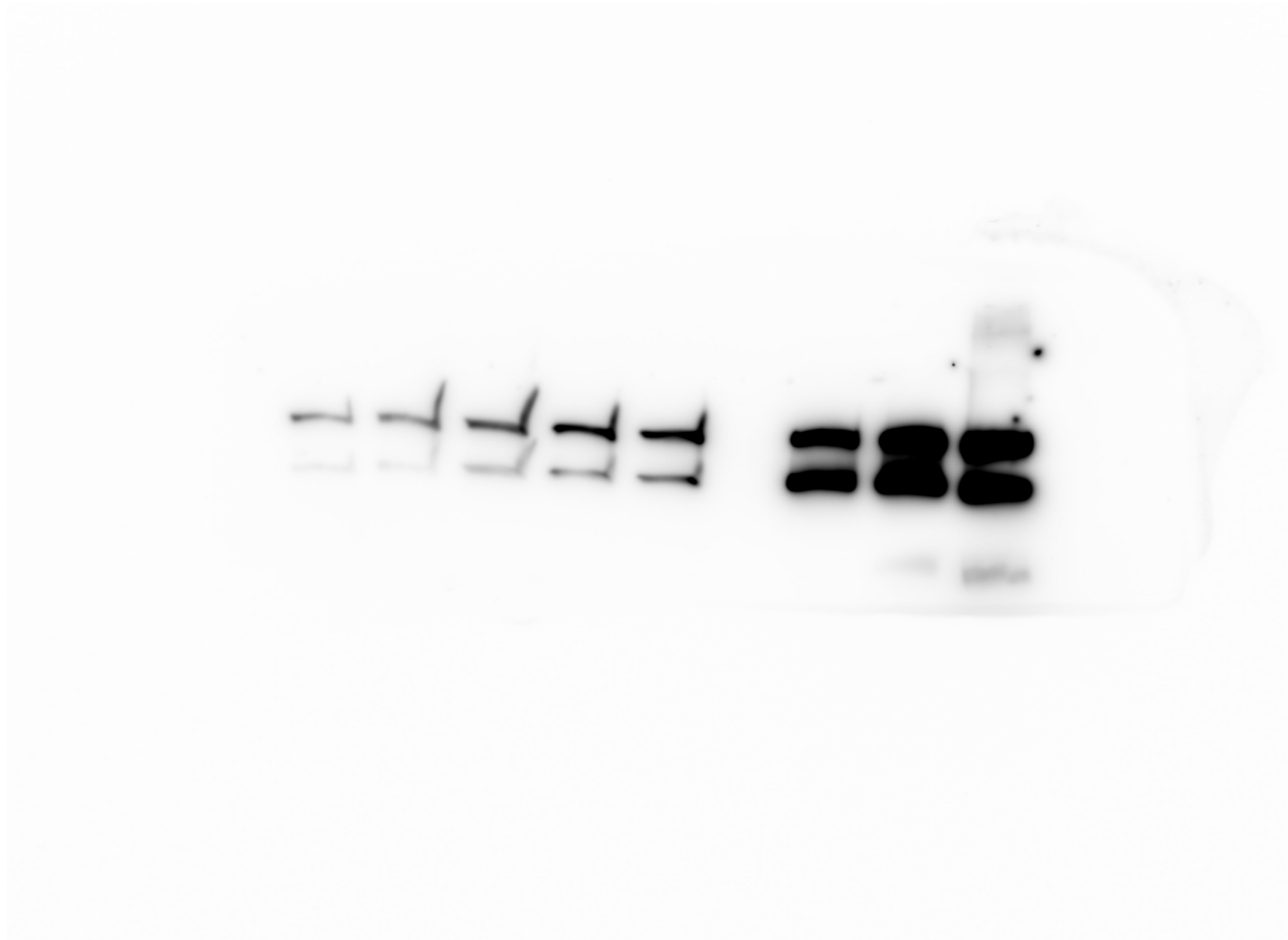

cterm DOCK11 T cells WB

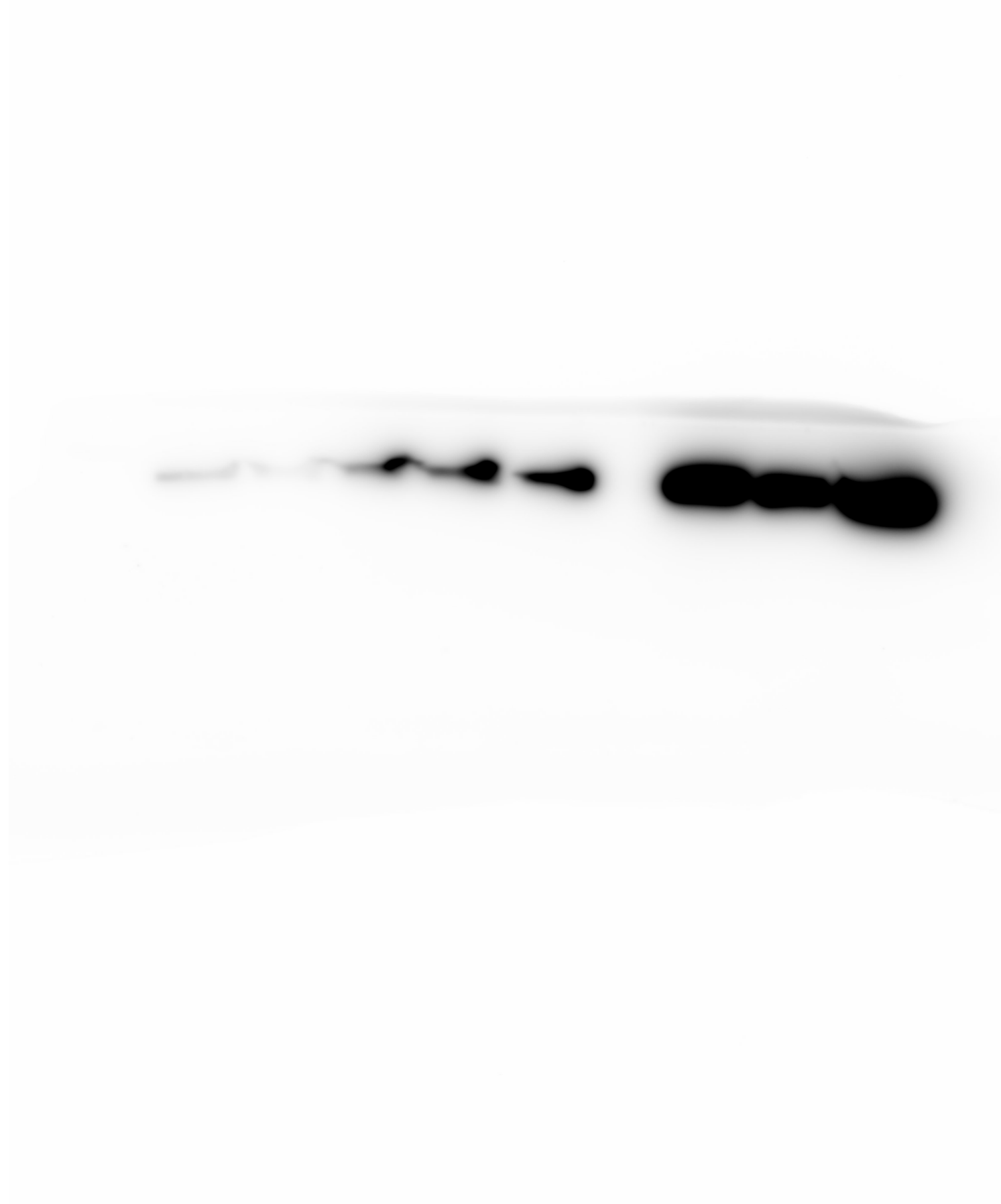

beta actin T cells WB

Supplement: SourceData F1 — is the source file for Fig. 1. [file jhi_20260053_sourcedataf1.pdf]
